# Comparison of High Flow Nasal Cannula and Continuous Positive Airway Pressure in COVID-19 Patients With Acute Respiratory Distress Syndrome in Critical Care Unit: A Randomized Control Study

**DOI:** 10.7759/cureus.45798

**Published:** 2023-09-22

**Authors:** Gunaseelan Mirunalini, Kuppusamy Anand, Anand Pushparani, Gunasri Kadirvelu

**Affiliations:** 1 Anesthesiology, SRM (Sri Ramaswamy Memorial) Medical College Hospital and Research Centre, Chennai, IND

**Keywords:** invasive mechanical ventilation, continuous positive airway pressure (cpap), high flow nasal cannula (hfnc), acute hypoxic respiratory failure, covid-19

## Abstract

Background and objective

Acute hypoxic respiratory failure in coronavirus disease 2019 (COVID-19) pneumonia has been treated with oxygen delivered by oxygen masks and non-invasive ventilation (NIV) with continuous positive airway pressure (CPAP), and more recently with high-flow nasal cannula (HFNC) devices. There is a paucity of randomized controlled trials to compare the efficacy of CPAP with HFNC in COVID-19 pneumonia. We conceptualized a randomized control study to compare the efficacy of HFNC and CPAP in reducing the need for invasive mechanical ventilation, estimation of mechanical ventilation-free days, and risk of intubation in COVID-19 patients with hypoxic respiratory failure.

Methodology

One hundred consecutive patients who satisfied the inclusion criteria were included in the trial. The patients were then randomly allocated to receive either CPAP or HFNC with settings as per the study protocol. The patients were deemed to have achieved the study endpoint when they were intubated due to any reason or successfully weaned from NIV to conventional oxygen therapies. The number of patients who required invasive ventilation and the number of invasive ventilation-free days were recorded and analyzed.

Results

Nineteen (38%) patients in the CPAP group and 30 (60%) patients in the HFNC group required invasive mechanical ventilation and the difference was statistically significant (p = 0.03, 95%CI: 0.1829-0.9129). The median number of days free of invasive mechanical ventilation in the CPAP group (median=5 (interquartile range (IQR(=5,6)) was more than in the HFNC group (median=4 (IQR=3,4)) and this difference was statistically significant (p<0.000). The secondary analysis of risk evaluation for intubation done using the Cox regression model showed no significant factors that could have contributed to intubation in the study population. The Kaplan-Meyer curve was used to express the probability of a patient getting intubated and the calculated hazard ratio was 2.29.

Conclusion

The administration of CPAP significantly reduced the intubation rate and prolonged invasive mechanical ventilation-free period in COVID-19 patients with hypoxic respiratory failure. We also inferred a two-fold increase in the risk of intubation in patients receiving HFNC compared to CPAP.

## Introduction

Acute hypoxic respiratory failure (AHRF) due to acute respiratory distress syndrome (ARDS) is an important cause of morbidity and mortality in the intensive care unit (ICU) in patients with severe acute respiratory syndrome coronavirus 2 (SARS‑CoV‑2) [1]. The pathophysiology of ARDS is extensive alveolar damage leading to ventilation-perfusion mismatch and hypoxemia. Improving oxygenation with invasive ventilation or non-invasive ventilation (NIV) is the cornerstone of treating hypoxemia in COVID-19-induced ARDS. High-flow nasal cannula (HFNC) and continuous positive airway pressure (CPAP) are commonly used to treat mild to moderate hypoxia in COVID-19 patients along with appropriate medical therapy with favorable outcomes [2].

Ever since its discovery in the 1980s, NIV eventually became the first-line intervention for some types of acute respiratory failure [3]. CPAP is a non-invasive treatment that reduces the work of breathing by unloading the respiratory muscles and providing a positive airway pressure thereby improving ventilation. In CPAP mode, mechanical ventilator support is administered using a nasal, oro-nasal, or full-face mask or a helmet [3]. There are several studies describing the advantages of CPAP in reducing the need for invasive mechanical ventilation in acute respiratory failure [4-6]. However, CPAP has a few complications like increased patient discomfort due to the tight-fitting face mask, nasal ulcerations due to dry air delivery, and pneumothorax [7].

HFNC is a non-invasive treatment modality capable of delivering a high flow of humidified warm oxygen through a nasal cannula [8]. HFNC has been postulated to work by generating positive end-expiratory pressure (PEEP), reducing anatomical dead space, maintaining a constant fraction of inspired oxygen (FiO2) with humidification, and correcting ventilation-perfusion mismatch seen in ARDS [9,10]. Since HFNC provides humidified air by using an air-oxygen blender and an active warming device, the side effects due to the administration of dry gas are completely eliminated. The advantages of HFNC as an early non-invasive treatment modality that can reduce the need for mechanical ventilation have been established [10,11]. The effectiveness of oxygenation by HFNC has been studied extensively in the last few years [8,12,13]. Still, HFNC has been only used as an alternate oxygen delivery device to conventional nasal cannulas, face masks, and non-rebreathing masks till recently [14,15]. During the COVID-19 pandemic, there was an increase in the use of HFNC in the management of ARDS.

A few cohort and retrospective observational studies comparing the outcomes of ARDS patients treated with CPAP and HFNC have been conducted and the results have been inconsistent with regard to treatment failure rates, intubation rates, 28-day mortality, and re-intubation rates [16-21].

Hence, we conceptualized a randomized control study to compare the efficacy of HFNC with CPAP in reducing the need for invasive mechanical ventilation and estimation of invasive ventilation-free days in COVID-19 patients with hypoxic respiratory failure.

The primary outcome of our study was to detect the number of COVID-19 patients with AHRF who require invasive mechanical ventilation after treatment with HFNC and CPAP. The secondary outcomes measured were the number of invasive ventilation-free days and the risk of intubation.

## Materials and methods

This was a single-center, open-labeled, parallel arm, 1:1 allocation, prospective, randomized controlled trial done on 100 patients with ARDS admitted to the critical care unit of a tertiary care center, SRM (Sri Ramaswamy Memorial) Medical College Hospital and Research Centre, Chennai, India, from October 2021 to March 2022. The trial was approved by the Institutional Ethical Committee of SRM Medical College Hospital and Research Centre (approval number: 2068/IEC/2021) and registered in the Clinical Trial Registry of India (CTRI/2021/04/032501). The study strictly adhered to the Declaration of Helsinki.

All consenting patients above 18 years of age who were COVID-19 positive as detected by reverse transcriptase-polymerase chain reaction (RT-PCR) with peripheral oxygen saturation of less than 92% with low flow oxygen therapy for 10 minutes, normal sensorium, stable hemodynamics, pH more than 7.2, absence of nasal pathology and partial pressure of oxygen (PaO2)/FiO2 ratio between 100-300 were included in the study. Patients who did not satisfy inclusion criteria or had pneumothorax, chronic obstructive pulmonary disease, epistaxis, and basal skull fracture were excluded from the study.

A total of 330 patients who were admitted with COVID-19-pneumonia-induced ARDS were evaluated and 100 patients who satisfied the inclusion criteria were included in the trial. The number of days with symptoms and the treatment modality instituted previously were not considered for inclusion. The patients were then randomly allocated into two groups, H and C, by computer-generated random number allotment by sealed envelope technique. Allocation concealment was done using a sealed envelope method by a person who was not involved in the intervention, data collecting, or data analysis procedure. Due to the nature of the intervention, blinding of patients and investigators was not possible. Patients were administered medications like steroids, heparin, antiviral drugs, and feeds as per standard institution protocol.

**Figure 1:**
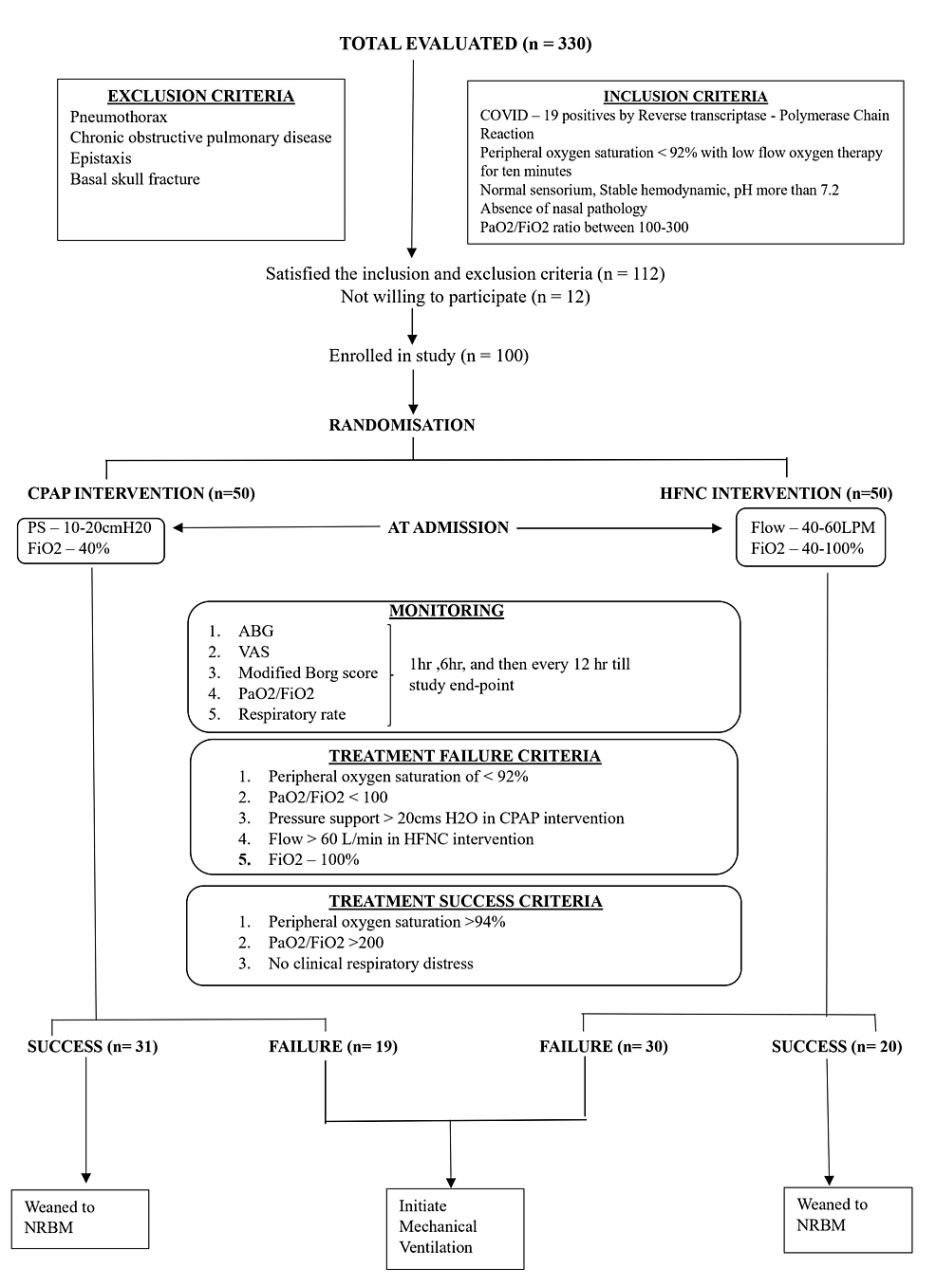
Flowchart depicting workflow and patient management PS: pressure support; Fi02: fractional concentration of inspired oxygen; ABG: arterial blood gas; VAS: visual analogue scale; NRBM: Non-rebreathing mask

The workflow and management are shown in Figure 1. Patients admitted in group H were administered oxygen by HFNC with initial settings of FiO2 40% with a flow rate of 40 l/min. Patients were monitored clinically by respiratory rate, increased work of breathing as assessed by overactive accessory respiratory muscles, and objectively by continuous pulse oximetry, arterial blood gas analysis at one hour, six hours, and thereafter every 12 hours. In the event of deterioration of respiratory parameters, FiO2 and flow rates were increased to a maximum of 100% and 60 l/min, respectively. Patients admitted to group C were administered CPAP with pressure support of 10 cm water. Pressure settings were titrated up to 20 cmH2O and oxygen flow was increased as per the attending anesthesiologist's orders to maintain saturation of peripheral oxygen (SpO2) above 92%. The patients were deemed to have achieved the study endpoint when they were intubated due to any reason or successfully weaned off from NIV. Treatment failure in either of the groups was defined as SpO2 <92%, PaO2/FiO2 ratio of <1 with maximum possible pressure flow, FiO2 in each group, and treatment with invasive mechanical ventilation. To obtain an adequate sample size to satisfactorily answer all the objectives of the study, the chi-square goodness of fit test was used to calculate the sample size of the study using G*Power 3.1.9.4. With reference to the study conducted by Demoule et al. [22], the sample size to determine a 30% reduction in the number of patients on HFNC requiring intubation, with 80% power and alpha error 0.05, the sample size was calculated to be 88. The total sample size was taken as 100 to account for dropouts, if any.

In order to analyze the normal distribution of the collected data, we used Kolmogorov-Smirnov test. GraphPad Prism (Dotmatics, Boston, Massachusetts) was used to perform the statistical analysis. We used unpaired t-test to compare continuous variables in the groups and the results were expressed as mean and standard deviation. The median with interquartile range was used to express discrete data and the Mann-Whitney U test was used to compare medians. Categorical variables were reported as percentages and Pearson's chi-square was used to determine statistical significance. We considered the difference between groups to be significant if p<0.05. The result of the primary analysis was provided as a p-value and 95% confidence interval. To analyze the secondary outcome of the risk of intubation, we used the Cox regression model including variables at ICU admission, such as demography of the patient, sequential organ failure assessment (SOFA) scale to assess the risk of mortality, modified Borg scale to grade dyspnea, and PaO2/FiO2 ratio. The time-to-event analysis for intubation was performed using Kaplan-Meyer curves and the p-value was estimated using a log-rank test.

## Results

**Figure 2:**
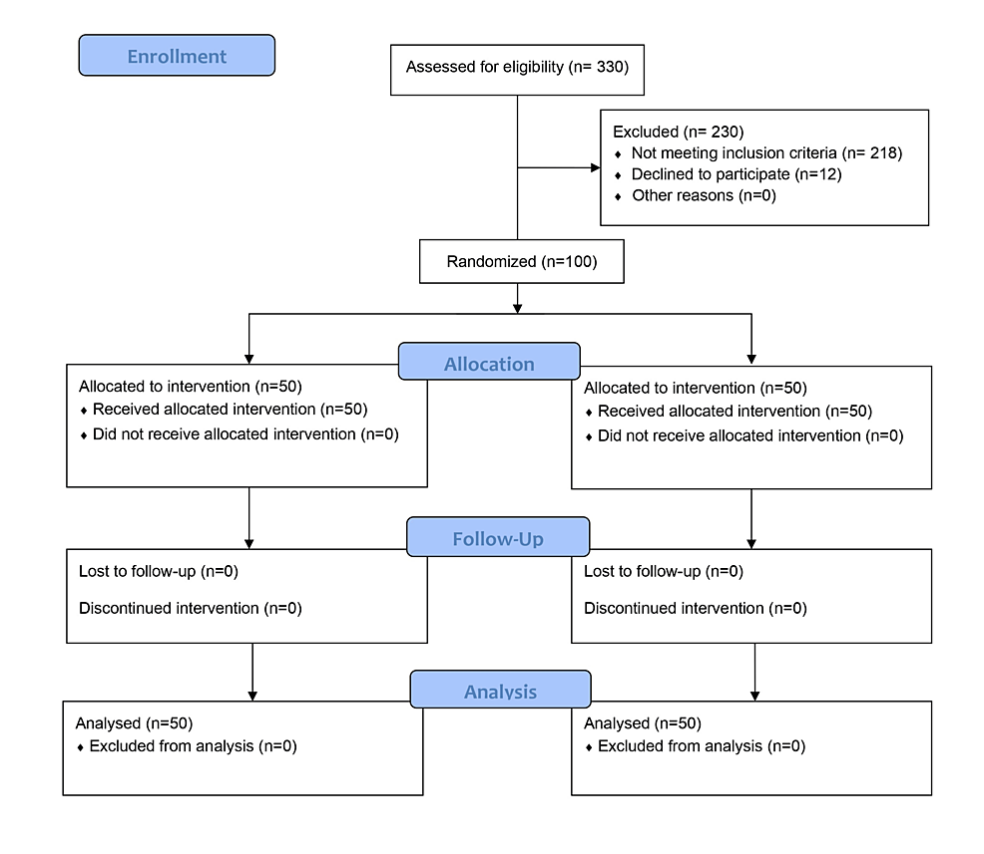
CONSORT flow diagram CONSORT: Consolidated Standards of Reporting Trials

A total of 330 patients were evaluated for the study and out of these, 100 patients were included in the study. Fifty patients were allotted to each group to receive the allotted intervention. The flow of the study population from evaluation to analysis is shown in Figure 2.

There was no crossover between the two treatment groups. All the patients were followed for a period of 28 days from the day of admission to the ICU. Both groups were statistically comparable with respect to demographic profile. The results are shown in Table 1.

**Table 1:** Demographic details and admission parameters BMI: body mass index; PaO2: partial pressure of oxygen; FiO2: fraction of inspired oxygen; SOFA: sequential organ failure assessment

Parameters	Group C	Group H	P value
Age (years)	57.92 ± 8.43	56.96 ± 8.11	0.56
Sex			0.42
Male (n)	26	22	
Female (n)	24	28	
BMI (kg/m2), mean±SD	28.02 ± 5.59	27.69 ± 5.83	0.77
Respiratory rate (per minute), median (IQR)	27.5 (26-28)	26 (27-28)	0.11
PaO2/Fi02 ratio, mean±SD	192.06 ± 42.77	193.3 ± 40.02	0.89
SOFA score, median (IQR)	5 (4-6)	5 (5-6)	0.89
Modified Borg scale, median (IQR)	3 (3-4)	3 (3-4)	0.17

The admission SOFA score, Borg score, respiratory rate, and PaO2/Fi02 ratio of the patients were collected to assess the severity of disease in the study participants. The median SOFA score in both groups was 5. The factors that contributed to the SOFA score in addition to the PaO2/Fi02 ratio were the increased serum urea and a mean arterial pressure of less than 70 mmHg but not requiring hemodynamic support. Table 1 shows the other admission characteristics of patients in both groups.

The primary outcome analysis showed that 19 (38%) patients in Group C and 30 (60%) patients in Group H required invasive mechanical ventilation. We conclude that more patients in Group H required intubation and this difference was statistically significant (p = 0.03, 95%CI: 0.1829-0.9129). The median number of days free of invasive mechanical ventilation in Group C (median=5 (interquartile range (IQR)5,6)) was more than in Group H (median=4 (IQR=3,4)), and this difference was statistically significant (p<0.000) (Table 2).

**Table 2:** Primary and secondary outcomes VAS: visual analogue score; PaO2: partial pressure of oxygen; FiO2: fraction of inspired oxygen * p<0.05 statistically significant

Outcomes	Group C	Group H	p-value
Primary Outcome			
No. of patients who needed intubation, n (%)	19 (38%)	30 (60%)	0.03*
Secondary Outcomes			
Period without intubation in days, median (IQR)	5 (5-6)	4 (3-4)	0.0001*
PaO2/FiO2 ratio, mean±SD			
1 hour	198.7 ± 39.7	197.6 ± 37.8	0.89
6 hours	211.9 ± 29.	201.8 ± 37.1	0.13
12 hours	219.3 ± 24.3	205.1 ± 37.9	0.03*
Respiratory rate (per minute), median (IQR)			
1 hour	22 (20-24)	23.5 (21-24)	0.04*
6 hours	21 (19-24)	22 (20-24)	0.66
12 hours	22 (19-24)	22 (19-24)	0.56
Modified Borg score, median (IQR)			
1 hour	2 (1-3)	3 (2-4)	0.002*
6 hours	2 (1-3)	3 (2-3)	0.002*
12 hours	1 (0-3)	3 (2-3)	0.003*
24 hours	1 (0-2)	2 (1-3)	<0.0001*
48 hours	0.5 (0-2)	2 (1-3)	0.003*
VAS scale for discomfort, mean ± SD			
1 hour	40.1 ± 14.8	42.8 ± 12.1	0.23
12 hours	56.8 ± 13.7	42.1 ± 11.8	<0.0001*
24 hours	56.9 ± 16.8	43.7 ± 16.7	0.0006*
48 hours	58.5 ± 15.7	42.1 ± 11.8	<0.0001*

The secondary analysis of risk evaluation for intubation done using Cox regression model showed no significant factors that could have contributed to intubation in the study population. The results are shown in Table 3.

**Table 3:** COX proportional hazard BMI: body mass index; SOFA: sequential organ failure assessment; PaO2: partial pressure of oxygen; FiO2: fraction of inspired oxygen

Variable	Relative risk	95% CI	p-value
Univariate COX regression			
Age	0.9523	[0.7082-1.2805]	0.7427
Sex	2.5695	[0.4255-15.5152]	0.2973
BMI	1.0543	[0.9254-1.2013]	0.4346
PaO2/FiO2 ratio	0.9975	[0.9638-1.0323]	0.8849
Respiratory rate	0.7525	[0.3408-1.6616]	0.4714
Modified Borg score	0.8287	[0.2391-2.8715]	0.7655
SOFA	0.9326	[0.2435-3.5716]	0.9188
Multivariate COX regression			
Age	0.9684	[0.9135-1.0265]	0.2798
Sex	0.8039	[0.4555-1.4186]	0.4512
BMI	1.0141	[0.9669-1.0636]	0.5643
PaO2/FiO2 ratio	1.0005	[0.9937-1.0075]	0.2915
Respiratory rate	0.8828	[0.7002-1.1130]	0.8762
Modified Borg score	1.0147	[0.6826-1.5083]	0.9425
SOFA	1.0713	[0.7297-1.5727]	0.7252

**Figure 3:**
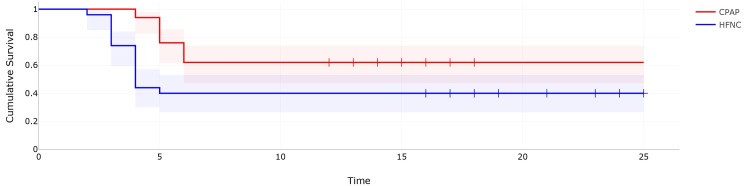
Kaplan-Meier survival curve CPAP: continuous positive airway pressure; HFNC: high-flow nasal cannula

The Kaplan-Meyer curve was used to express the probability of patient survival in both groups over time and the calculated hazard ratio was 2.29 (Figure 3).

There was a two-fold increase in the risk of intubation in patients receiving HFNC compared to CPAP and this difference was statistically significant (p = 0.001) when tested using log-rank test.

**Figure 4:**
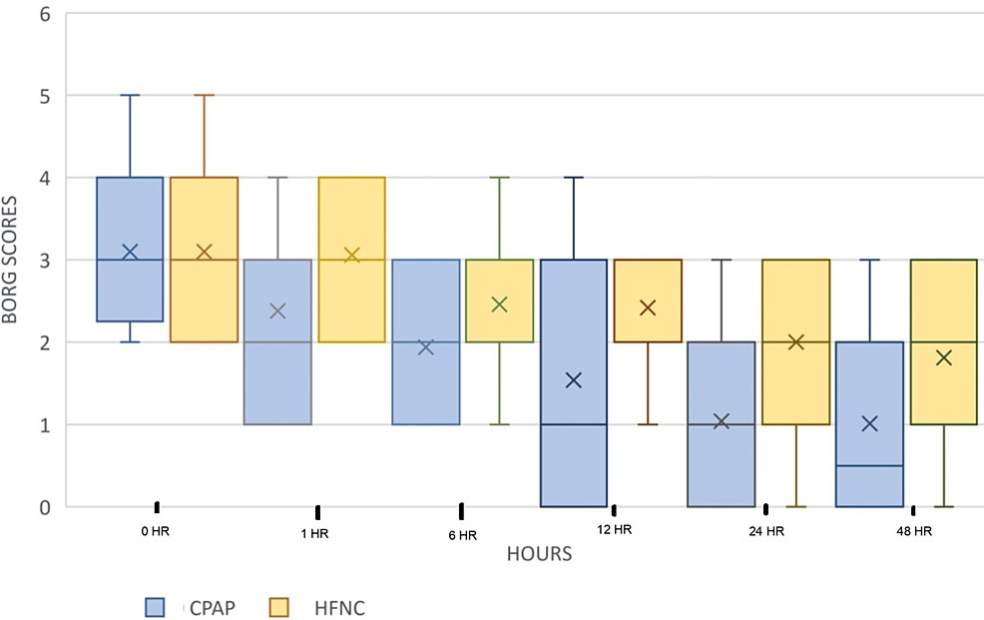
Trend of modified Borg score over time HR: hour; CPAP: continuous positive airway pressure; HFNC: high-flow nasal cannula

**Figure 5:**
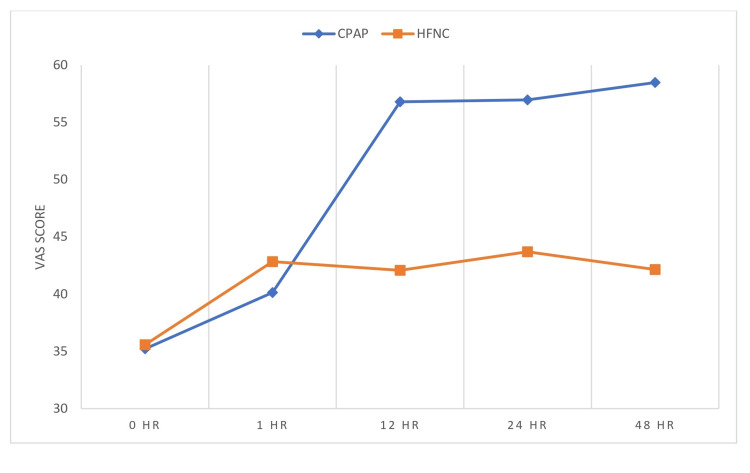
Mean of VAS score of patient discomfort HR: hour; CPAP: continuous positive airway pressure; HFNC: high-flow nasal cannula; VAS: visual analogue scale

**Figure 6:**
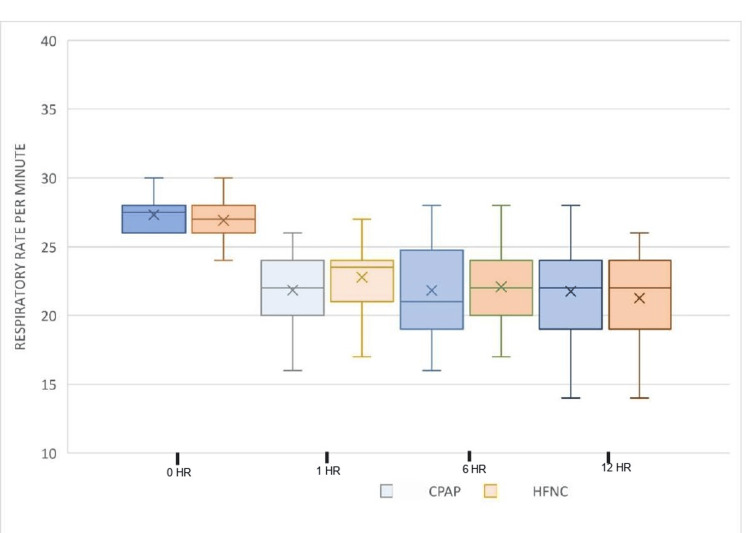
Comparison of respiratory rate over time HR: hour; CPAP: continuous positive airway pressure; HFNC: high-flow nasal cannula

**Figure 7:**
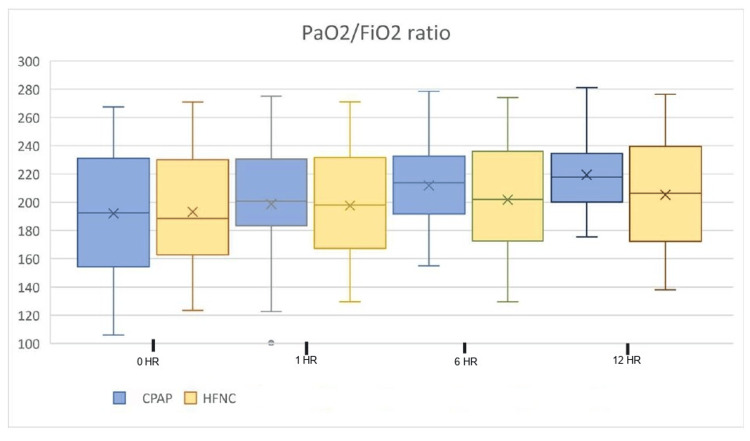
PaO2/FiO2 ratio HR: hour; PaO2: partial pressure of oxygen; FiO2: fraction of inspired oxygen; CPAP: continuous positive airway pressure; HFNC: high-flow nasal cannula

Other outcome parameters measured were PaO2/FiO2 ratio and respiratory rate during the first hour and thereafter every sixth hour. The modified Borg score for dyspnea and VAS to assess overall comfort while using oxygen delivery devices for the first 48 hours after initializing the treatment were also observed. We observed a significant reduction in respiratory rate in the Group C in the first hour after initiation of the treatment and improvement in PaO2/FiO2 ratio at the 12th hour post intervention. On analyzing the modified Borg score for dyspnea, we found that there was a reduction in the dyspnea scale in both groups of patients after initiation of the respective intervention. The difference between the groups was statistically significant with the patients in Group C showing greater alleviation of dyspnea. Higher VAS scores in patients on CPAP, however, indicated a significantly high discomfort level in patients on CPAP. The results are shown in Figures 4-7. There were no incidences of complications like pneumothorax, perinasal ulcerations, epistaxis, and sore throat in either groups.

## Discussion

Although invasive mechanical ventilation remains the gold standard for improving oxygenation, it is associated with side effects like barotrauma, ventilator-induced lung injury, and ventilator-associated pneumonia (VAP) [23]. NIV has gained popularity as they have been shown to deliver oxygen with fewer complications. CPAP helps in actively reducing alveolar edema, preserving surfactant, and recruiting collapsed alveoli [24]. HFNC on the other hand reduces airway resistance, improves lung compliance and mucociliary function, reduces nasopharyngeal dead space, and maintains positive airway pressure [8]. While both CPAP and HFNC help to improve ventilation, CPAP with facemask interface has been shown to be associated with more patient discomfort and aerophagia [25,26]. This has led to increased use of HFNC, the efficacy of which has been evaluated in a few cohort and observational studies. However, the effect of HFNC on the intubation rates in patients with COVID-19-induced ARDS is still not evaluated in randomized controlled trials. Hence, we devised a randomized controlled study to compare the efficacy of CPAP and HFNC in reducing the need for intubation in patients affected with COVID-19.

We found that patients on CPAP had lower intubation rates than HFNC and the difference was statistically significant. Duan et al. conducted a similar study using facemask CPAP and compared the intubation rates in patients with HFNC [19]. They concluded that administration of CPAP reduced the need for endotracheal intubations (15%) more than HFNC (17%) but the difference was not statistically significant. Zhao et al. conducted a study in a pediatric population with respiratory failure comparing the use of HFNC and CPAP and concluded that HFNC use was associated with twice the rate of intubation [27]. This result is also supported by a study conducted by Gaulton et al. where 52.4% of COVID-19-induced ARDS patients in the HFNC group and only 17.7% of patients in the CPAP group required intubation [28]. Another study conducted by Grieco et al. also supports our conclusion in which 51% of patients in the HFNC group and 30% of patients in the CPAP group needed intubation [29]. It is important to note that the results of the above-mentioned studies were obtained by comparing helmet CPAP with HFNC, which is in contrast to our study where we used facemask CPAP as an interface. The PaO2/FiO2 ratios in the CPAP group were higher than in the HFNC group indicating better oxygenation due to greater PEEP.

The median ventilator-free period observed in our study was significantly longer in the CPAP group compared to the HFNC group. While both the CPAP and HFNC have been shown to generate PEEP and help in better oxygenation, the positive pressure generated by HFNC is lesser and varies compared to CPAP [7]. This explains better oxygenation as demonstrated by the higher PaO2/FiO2 ratio seen in the CPAP group, thereby reducing the need for invasive ventilation. Patients who were administered CPAP demonstrated statistically significant reduction of respiratory rate and modified Borg scale in our study. Gough et al. and Kang et al. showed that early use of HFNC led to a delay in intubation [25,26]. However, this was mainly attributed to the increased acceptance of HFNC by the patients.

The main strength of our study was that it was conducted as a prospective randomized controlled study, which could provide the highest level of clinical evidence. Also, when analyzing data there were no missing data and no crossovers between the groups which strengthened the observed effect size. 

There are a few limitations to our study. We did not consider the duration of symptoms and treatment given before admission at enrolment. This could have provided more valuable information regarding the primary outcome of our study. Secondly, we only included patients with moderate to mild ARDS in our study, which could have affected the outcome. Third, we did not follow up on the outcome of intubated patients to look for mortality rates as this was beyond the scope of our study. This data could have provided more evidence on the overall outcome of patients treated with HFNC and CPAP in ARDS.

## Conclusions

We conclude that the administration of CPAP significantly reduced the intubation rate and prolonged invasive mechanical ventilation-free period in COVID-19 patients with hypoxic respiratory failure. There was a two-fold increase in the risk of intubation in patients receiving HFNC compared to CPAP.
